# Comparative analysis of gastric cancer risk attribution (1990-2021) and 2050 burden projection in China, Japan, and South Korea: an age-period-cohort modeling approach based on the Global Burden of Disease 2021 study

**DOI:** 10.3389/fonc.2025.1680684

**Published:** 2026-01-21

**Authors:** Tao Jiang, Liming Tan, Kehang Dai

**Affiliations:** 1 Department of Nuclear Medicine, Hunan University of Medicine General Hospital, Huaihua, Hunan, China; 2 Department of Pharmacy, Huaihua Central Hospital (Huaihua Cancer Hospital), Huaihua, Hunan, China; 3 Department of Pharmacy, Hunan University of Medicine General Hospital, Huaihua, Hunan, China; 4 Department of Gastrointestinal, Chongqing University Three Gorges Hospital, Chongqing, China

**Keywords:** Bayesian Age-Period-Cohort, China, disability-adjusted life years, gastric cancer, Global Burden of Disease, Japan, South Korea

## Abstract

**Background:**

Gastric cancer (GC) is the fifth most common cancer and fourth leading cause of cancer-related death globally, with a particularly high burden in East Asia. Significant differences exist among China, Japan, and South Korea in terms of risk factor exposure, screening practices, and demographic shifts, yet existing research lacks cross-national comparisons of long-term trends and quantitative analyses of policy effectiveness; this study aims to systematically analyze the spatiotemporal evolution of GC burden in these three countries from 1990 to 2050 by integrating the Global Burden of Disease (GBD) 2021 database with the Bayesian Age-Period-Cohort (BAPC) model to provide evidence for Asia-Pacific prevention and control strategies.

**Methods:**

We extracted data on key GC epidemiological indicators—including age-standardized incidence rate (ASIR), age-standardized mortality rate (ASMR), and age-standardized disability-adjusted life years rate (ASDR)—as well as relevant risk factor data from 1990 to 2021 using the GBD 2021 database. An enhanced Age-Period-Cohort (APC) analytical framework was adopted, and log-linear models were constructed to quantify the independent impacts of age, period, and cohort effects on GC burden. The population attributable fraction (PAF) method was applied to estimate the proportion of DALYs attributable to modifiable risk factors such as smoking and high-sodium diets. For trend projection (2022–2050), the BAPC model was utilized, forming a comprehensive analytical chain that spanned data extraction, effect decomposition, and future burden forecasting.

**Results:**

From 1990 to 2021, age-standardized incidence rate (ASIR), age-standardized mortality rate (ASMR), and age-standardized disability-adjusted life years rate (ASDR) of GC declined significantly across China, Japan, and South Korea. The absolute burden trends differed among the three countries: new GC cases in China increased from 300,000 in 1990 to 612,000 in 2021, with annual deaths reaching 445,000; Japan and South Korea had 9% and 7% reductions in new cases, respectively, along with substantial declines in mortality. Risk attribution analysis showed that smoking was the primary factor associated with GC burden among males in China, while high-sodium diets were the dominant associated factor in Japan and South Korea. South Korean women aged 20–49 had a higher incidence rate than their male peers (relative risk [RR] = 1.23). Decomposition analysis identified adults aged ≥65 years as the main burden group: this age group contributed 60%–70% of ASIR and ASMR in China, 55%–65% in Japan, and 50%–60% in South Korea. After 2000, the contribution of period effects to ASMR continued to decrease across the three countries. Later birth cohorts (post-1970) had significantly reduced GC risk: compared with pre-1950 cohorts, post-1970 cohorts in China had a 20% lower ASIR (reflected in a 16% lower risk among 30–34-year-olds), and South Korea’s post-1970 cohorts had a 30% reduction (manifested as a 58% lower risk in the 50–54 age group). Projections to 2050 indicated that ASIR and ASMR will continue to decline across the three countries, with China’s ASIR ≈17/100,000 and ASMR ≈8/100,000, and Japan and South Korea’s ASIR ≈8–9/100,000. China’s absolute GC burden will remain higher than that of Japan and South Korea, driven by population aging and the persistent impacts of smoking and high-sodium diets.

**Conclusions:**

This study finds that from 1990 to 2021, ASIR, ASMR, and ASDR of GC in China, Japan, and South Korea showed a marked decline, yet absolute disease burdens differed. China’s burden remained heavy due to its large population and rapid aging, while Japan and South Korea made substantive progress via efficient screening. Risk attribution analysis revealed smoking as China’s main risk factor and high-salt diets as more impactful in Japan and South Korea. Projections suggest a continued decline in disease burden in all three countries by 2050.

## 1 Introduction

Gastric cancer (GC) continues to pose a major global health challenge, standing fifth in incidence and fourth in mortality worldwide (1). Data from the Global Burden of Disease Study 2021 (GBD 2021) shows that GC caused roughly 954,000 deaths globally in 2021, with East Asia bearing a disproportionate share of this burden (2). Even though China, Japan, and South Korea account for just 22% of the world’s population, they represent over 60% of global GC cases (2). This geographic variation highlights how region-specific risk factors, screening practices, and demographic shifts shape the disease burden. In 2021, East Asia reported 624,688 GC cases—50.8% of the 1.23 million global total. China alone recorded 445,000 GC deaths, making up 46.6% of global GC mortality (2).

Trends in GC burden across China, Japan, and South Korea reflect the complex interplay between public health policies and socioeconomic factors. Japan’s national Helicobacter pylori screening and eradication program, launched in 2013, is associated with a 40% lower GC risk in treated groups, contributing to sustained declines in the age-standardized mortality ratio (ASMR) (3). Similarly, South Korea’s National Cancer Screening Program (NCSP)—which offers biennial endoscopies to adults aged ≥40—has helped achieve early-stage diagnosis rates over 65% and five-year survival rates above 75%, the highest globally (4). In contrast, China faces ongoing challenges: rural areas have screening coverage below 30%, and dietary risk factors are becoming more prevalent. High sodium intake (10–12 g/day) accounts for 28.7% of GC related disability-adjusted life years (DALYs) in China, alongside H. pylori infection rates over 50% among adults aged ≥60 (5).

Current research on GC epidemiology in East Asia has several key limitations. First, most studies use cross-sectional designs or short-term trend analyses, making it hard to separate the distinct impacts of aging, birth cohort exposures, and period-specific interventions. For example, while Japan’s declining ASMR is well-documented, the relative contributions of dietary improvements (cohort effect) and endoscopic screening (period effect) have not been fully quantified (6). Second, most studies focus on single countries, which limits direct comparisons of policy effectiveness across nations (7). Third, risk attribution analyses often overlook regional differences in risk factor distributions—such as high-salt kimchi consumption in South Korea compared to tobacco use prevalence in Japan (27.5% among men) (8). Finally, projections of future GC burden fail to fully integrate demographic changes—including rapid population aging in East Asia (e.g., the share of adults aged ≥65 is expected to rise from 14% in China to 36% in Japan by 2050)—with cancer epidemiology, which limits their value for policy planning (9).

This study innovatively integrates a Bayesian Age-Period-Cohort (BAPC) prediction model with GBD demographic decomposition methods to systematically analyze the spatiotemporal evolution of GC burden in China, Japan, and South Korea from 1990 to 2050. Using the GBD 2021 database, we address three core research questions:

1. How do cohort effects explain the forward shift in peak incidence age among Korean males (50 years) versus the delayed peak (>70 years) in Chinese males?

Japanese studies indicate that post-1945 birth cohorts exhibit peak incidence 10–15 years earlier than pre-1930 cohorts due to reduced H. pylori (Hp) infection rates and screening accessibility (10). In contrast, Chinese cohorts born in 1950–1970 show significant peak delay, attributable to high Hp infection rates (40%-50%) and delayed screening implementation (11).

2. What is the contribution of screening policies to mortality decline under period effects?

Following Korea’s NCSP, GC mortality decreased by 3.2% annually, significantly surpassing China’s rate (1.8%) (11). Japan achieved a 2.5% annual reduction in incidence through Hp eradication (80% coverage), demonstrating synergistic efficacy of primary and secondary prevention (3).

3. How do interactions among risk factors (e.g., high-salt diet, smoking) drive regional disparities in disease burden?

In China, daily salt intake (10-12g) exceeds WHO recommendations (5g) and synergizes with Hp infection, increasing GC risk (OR = 2.17) (12). Korea’s salt-reduction initiatives (8g/day) combined with Hp eradication reduced risk by 34% (13).

This research provides evidence to optimize GC control strategies in the Asia-Pacific region, particularly informing screening policy refinement and Hp eradication scale-up in China. By integrating BAPC’s dynamic forecasting with GBD’s systematic decomposition, we address critical gaps in cross-national comparative research and offer empirical insights from China’s perspective for global cancer control.

## 2 Methods

### 2.1 Data sources and standardization

Study data were retrieved from the publicly available GBD 2021 database. We systematically collected epidemiological metrics for GC (ICD-10 codes C16.0-C16.9) in China, Japan, and South Korea between 1990 and 2021, including age-standardized incidence rate (ASIR), ASMR, ASDR, and their corresponding 95% uncertainty intervals (UI). Data were stratified by country and sex, and structured datasets were obtained via the GBD Results Visualization platform (https://vizhub.healthdata.org/gbd-results/). To identify drivers of the disease burden, we also extracted GBD data on GC related risk factors (e.g., Helicobacter pylori infection prevalence, high-sodium diet exposure, smoking prevalence). All metrics were age-standardized to mitigate confounding effects from differences in population age structure.

### 2.2 Age-period-cohort model specification and burden decomposition

To analyze spatiotemporal heterogeneity in GC burden and its drivers, we employed an enhanced APC framework. A log-linear model was constructed with age-standardized rate (ASR) as the dependent variable:


ln(ASRijk)=μ+αi+βj+γk+ϵijk

ln(ASR*_ijk_*): The natural logarithm of the ASR for the *i*​-th age group, *j*-th period, and *k*-th birth cohort. *μ*: The intercept term (overall mean effect). *α_i_*: The effect of the *i-*th 5-year age group (*i* = 1,2,…,20, corresponding to age groups 0–4 years to 95–99 years). *βj*: The effect of the *j-*th 5-year period (*j* = 1,2,…,5, corresponding to periods 1992–1996 to 2017-2021). *γk*: The effect of the *k*-th birth cohort (*k* = 1,2,…,25, corresponding to birth cohorts 1895–1899 to 2015-2019). *ϵijk*: The random error term (assumed to follow a normal distribution: *ϵijk*∼N(0,σ2)) (14).

Parameter estimation was carried out using the APC Web Tool (https://analysistools.cancer.gov/apc/) under identifiability constraints, with 2002–2006 designated as the reference period and the 1955–1959 birth cohort as the reference group.

Model decomposition consisted of two key components: period effect analysis, which quantified net drift (overall annual percentage change) and local drift (age-specific annual changes) to characterize temporal trends in GC burden and variations among different age groups; and cohort effect analysis, which derived longitudinal age curves (reference-cohort-adjusted age-specific rates) and relative risk ratios (RR) to examine intergenerational risk disparities across birth cohorts—quantifying the long-term impact of historical exposure patterns on disease burden. This integrated analytical strategy synthesizes temporal dynamics and generational effects, providing a multidimensional lens to understand the drivers underlying epidemiological transitions in GC.

Further integrating GBD risk factor data, we quantified the relative contribution of each factor to burden variations using the attributable fraction formula:


$$\text{Contribution Rate}\;(\%)=\frac{\text{Factor Effect Value}}{\sum \text{All Factor Effect Values}}\times 100$$

where Factor Effect Values include age, period, and cohort effect values derived from APC modeling; and population attributable fractions (PAF) from risk factor attribution analysis (15).

### 2.3 Risk factor attribution analysis and cohort linkage

Leveraging quantified GC risk factor data from the GBD database, we assessed the proportional attribution of each factor to DALYs using PAF, calculated as:


$$\text{PAF}=\frac{\sum \left(\text{Pi}\times \left(\text{RRi}-1\right)\right)}{1+\sum \left(\text{Pi}\times \left(\text{RRi}-1\right)\right)}$$

where Pi denotes the prevalence of risk factor i (e.g., Helicobacter pylori infection rate) and RRi represents the relative risk. We conducted linkage analysis between cohort effects derived from APC modeling and risk factor PAF (15). Consistency in intergenerational trends was verified through Pearson correlation testing (significance threshold: P<0.05), with focused cross-national comparison of risk factor contribution rates among China, Japan, and South Korea (e.g., examining whether high-sodium diet exposure in Japan’s post-war cohorts significantly exceeded that in contemporaneous Chinese cohorts).

### 2.4 Trend quantification and validation

Long-term trends in GC burden were quantified using the estimated annual percentage change (EAPC), calculated as:


EAPC=100×(exp(β)−1)

where β is the regression coefficient from the log-linear model of ASR. Trend significance was determined as: significant increase if EAPC > 0 with lower 95% confidence interval (CI) limit > 0; significant decrease if EAPC< 0 with upper 95% CI limit< 0; otherwise stable. To validate decomposition reliability, we performed cross-validation of cohort effects from APC modeling against intergenerational exposure trends in risk factor attribution analysis. We further assessed predictive accuracy improvement through the BAPC using the prediction interval coverage (PIC) metric after incorporating decomposition drivers.

### 2.5 Bayesian age-period-cohort projection model

We implemented a nested Laplace approximation model via the R package “BAPC” (v4.4.2) to project GC burden from 2022–2035 (16). Second-order random walk priors were applied to age, period, and cohort effects to ensure trend smoothness. Before projection, model calibration was evaluated through back-testing against historical data (1990–2021), generating median predictions with 95% credible intervals (CrI) that integrate prior distributions and data likelihood.

### 2.6 Statistical software and visualization

Data analysis was performed using R (v4.4.2) with packages including “BAPC” and “ggplot2” (16), where APC effects were visualized through the JD_GBDR (V2.22, Jingding Medical Technology Co., Ltd.) while projection trends were plotted using the “forecast” R package; decomposition results were presented via stacked area plots displaying cumulative driver contribution rates and heatmaps illustrating cross-national factor importance variations.

### 2.7 Ethics and data compliance

This study strictly adhered to the data usage guidelines of the Declaration of Helsinki. All analyses utilized publicly available, aggregated and de-identified data from the GBD study, which exempts the research from ethics committee review and individual patient informed consent requirements. Analytical tools were obtained under academic licensing agreements, ensuring compliance with publication ethics standards.

## 3 Results

### 3.1 Overall trends in gastric cancer burden and 2021 age-sex distribution

#### 3.1.1 Burden evolution from 1990 to 2021

**Figure 1:**
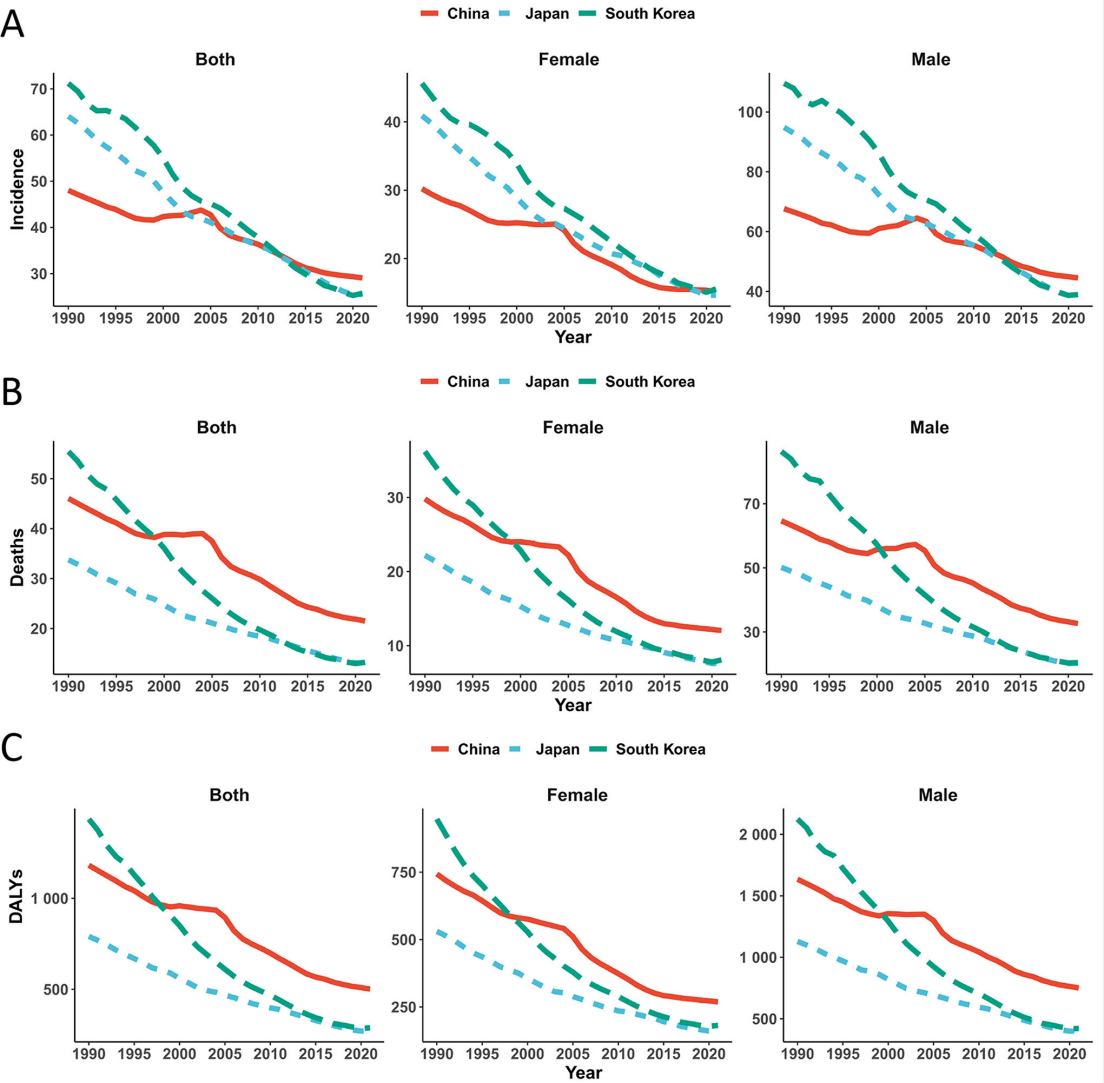
Age-standardized incidence **(A)**, mortality **(B)**, and DALYs **(C)** rates (per 100,000 population) of gastric cancer in China, Japan, and South Korea (1990–2021), stratified by sex. DALYs, disability-adjusted life years.

In 2021, new GC cases reached 612,000 in China, 99,000 in Japan, and 24,000 in South Korea (**Table 1**, **Supplementary Figure 1**). Compared to 1990, China saw a ~50% increase while South Korea increased by ~7%, contrasting with Japan’s ~9% reduction. Significant declines were observed in ASIR across all three nations, with males exhibiting higher rates than females but experiencing steeper reductions: South Korean males demonstrated the sharpest decline (109.56 to 38.98 per 100,000), followed by Japanese males (94.85 to 38.77 per 100,000), and Chinese males (67.64 to 44.48 per 100,000) (**Figure 1**). The accelerated decline in South Korea (-3.54%), moderate pace in Japan (-2.98%), and slower but significant reduction in China (-1.64%) were confirmed by EAPC (**Table 1**).

**Table 1:** Incidence, mortality, and DALYs of gastric cancer in China, Japan, and South Korea in 1990 and 2021, with estimated annual percentage changes from 1990 to 2021.

Characteristics	1990						2021						EAPC (1990-2021)		
	Incidence cases per 10,000 (95% UI)	Deaths cases per 10,000 (95% UI)	DALYs cases per 10,000 (95% UI)	ASIR per 100,000 (95% UI)	ASMR per 100,000 (95% UI)	ASDR per 100,000 (95% UI)	Incidence cases per 10,000 (95% UI)	Deaths cases per 10,000 (95% UI)	DALYs cases per 10,000 (95% UI)	ASIR per 100,000 (95% UI)	ASMR per 100,000 (95% UI)	ASDR per 100,000 (95% UI)	ASIR (95% CI)	ASMR (95% CI)	ASDR (95% CI)
China	40.75(33.76,47.76)	37.41(31.09,44.23)	1077.35(885.10,1263.89)	48.03(40.21,56.69)	46.05(38.88,54.43)	1181.61(978.38,1390.89)	61.18(47.20,76.56)	44.50(34.47,55.58)	1064.21(822.21,1338.38)	29.05(22.42,36.20)	21.51(16.66,26.61)	501.26(387.29,627.98)	-1.64(-1.82,-1.47)	-2.54(-2.76,-2.32)	-2.90(-3.09,-2.70)
Man	27.86(20.82,34.66)	25.16(18.82,31.44)	739.93(545.03,925.25)	67.64(51.71,83.67)	64.67(49.60,79.93)	1634.85(1218.61,2045.07)	44.64(32.59,58.93)	31.48(23.07,41.87)	774.04(563.43,1036.51)	44.48(32.18,58.38)	32.61(23.61,42.80)	750.39(550.90,997.91)	-1.28(-1.47,-1.10)	-2.20(-2.43,-1.97)	-2.56(-2.75,-2.36)
Female	12.89(10.52,15.77)	12.25(10.07,14.97)	337.42(273.45,416.06)	30.22(24.80,36.89)	29.81(24.69,36.43)	743.14(605.54,913.28)	16.54(12.77,20.81)	13.02(10.05,16.36)	290.18(225.17,367.94)	15.23(11.77,19.16)	12.02(9.29,15.10)	268.83(208.91,340.98)	-2.43(-2.63,-2.24)	-3.21(-3.45,-2.97)	-3.60(-3.83,-3.37)
Japan	10.83(10.31,11.15)	5.61(5.29,5.79)	133.26(128.36,136.75)	64.05(60.74,66.00)	33.76(31.64,34.94)	790.75(759.64,811.78)	9.90(8.53,10.67)	5.80(4.90,6.30)	92.52(81.59,98.44)	25.54(23.04,26.96)	13.20(11.67,14.04)	270.17(248.93,282.24)	-2.98(-3.05,-2.92)	-3.05(-3.10,-3.00)	-3.48(-3.54,-3.43)
Man	6.98(6.74,7.17)	3.51(3.39,3.62)	85.20(82.57,87.39)	94.85(91.32,97.59)	50.13(48.01,51.79)	1128.94(1092.71,1158.55)	6.54(5.91,6.88)	3.67(3.31,3.87)	61.96(57.25,64.86)	38.77(35.83,40.51)	20.26(18.58,21.26)	399.25(375.11,416.15)	-2.92(-3.00,-2.84)	-2.97(-3.03,-2.91)	-3.38(-3.44,-3.31)
Female	3.85(3.53,4.02)	2.10(1.90,2.20)	48.05(45.00,49.66)	40.90(37.65,42.67)	22.19(20.07,23.28)	530.36(498.75,547.42)	3.37(2.56,3.83)	2.13(1.58,2.46)	30.56(24.43,34.06)	14.66(12.25,15.99)	7.64(6.23,8.45)	160.33(141.01,171.15)	-3.32(-3.38,-3.26)	-3.45(-3.51,-3.39)	-3.89(-3.95,-3.84)
South Korea	2.22(1.68,2.51)	1.63(1.25,1.83)	48.91(35.62,54.96)	71.18(55.99,80.71)	55.44(44.07,62.45)	1435.39(1091.49,1613.83)	2.37(1.97,2.98)	1.23(1.01,1.53)	26.01(21.85,33.04)	25.76(21.53,32.40)	13.26(10.91,16.49)	288.82(242.93,366.07)	-3.54(-3.68,-3.41)	-5.02(-5.18,-4.86)	-5.46(-5.59,-5.33)
Man	1.41(0.95,1.63)	1.02(0.70,1.17)	30.88(20.45,35.54)	109.56(78.14,128.02)	86.29(62.78,102.05)	2123.84(1468.42,2455.30)	1.59(1.30,2.15)	0.80(0.65,1.08)	17.63(14.41,23.95)	38.98(31.66,52.47)	20.50(16.51,27.32)	421.60(344.20,568.98)	-3.62(-3.77,-3.47)	-5.05(-5.19,-4.90)	-5.54(-5.67,-5.42)
Female	0.80(0.63,0.97)	0.61(0.49,0.73)	18.03(14.04,21.52)	45.63(36.34,54.94)	36.20(29.28,43.60)	946.92(749.11,1128.55)	0.78(0.58,0.99)	0.43(0.31,0.55)	8.38(6.50,10.53)	15.57(11.94,19.69)	8.08(6.03,10.28)	181.94(143.40,227.76)	-3.71(-3.83,-3.60)	-5.24(-5.42,-5.06)	-5.54(-5.68,-5.39)

GBD, Global Burden of Disease; ASIR, age-standardized incidence rate; ASMR, age-standardized mortality rate; ASDR, age-standardized DALY rate; DALYs, disability-adjusted life-years; EAPC, estimated annual percentage change; UI, uncertainty interval; CI, confidence interval.

Despite rising death counts in Japan (increasing to 58,000) and China (445,000), South Korea recorded decreased mortality (12,000 deaths) (**Table 1**, **Supplementary Figure 1**). Male mortality consistently exceeded female rates. Crucially, ASMR declined significantly in all countries: South Korean males showed the most pronounced improvement (86.29 to 20.50 per 100,000), trailed by Japanese males (50.13 to 20.26 per 100,000) and Chinese males (64.67 to 32.61 per 100,000) (**Figure 1**). EAPC analysis revealed South Korea’s rapid progress (-5.02), Japan’s intermediate decline (-3.05), and China’s slower yet significant decrease (-2.54) (**Table 1**).

DALYs attributable to GC decreased substantially in Japan (to 925,000) and South Korea (260,000), while China showed a marginal reduction (to 10.642 million) (**Table 1**, **Supplementary Figure 1**). China exhibited significant gender divergence: male DALYs increased (7.40 to 7.74 million) whereas female DALYs decreased (3.37 to 2.90 million). ASDR declined significantly across all nations, with South Korean males experiencing the greatest reduction (2,123.84 to 421.60 per 100,000) (**Figure 1**). EAPC confirmed South Korea’s accelerated decline (-5.46), Japan’s intermediate pace (-3.48), and China’s more gradual but significant improvement (-2.90) (**Table 1**).

#### 3.1.2 Age-sex distribution patterns of gastric cancer burden in 2021

**Figure 2:**
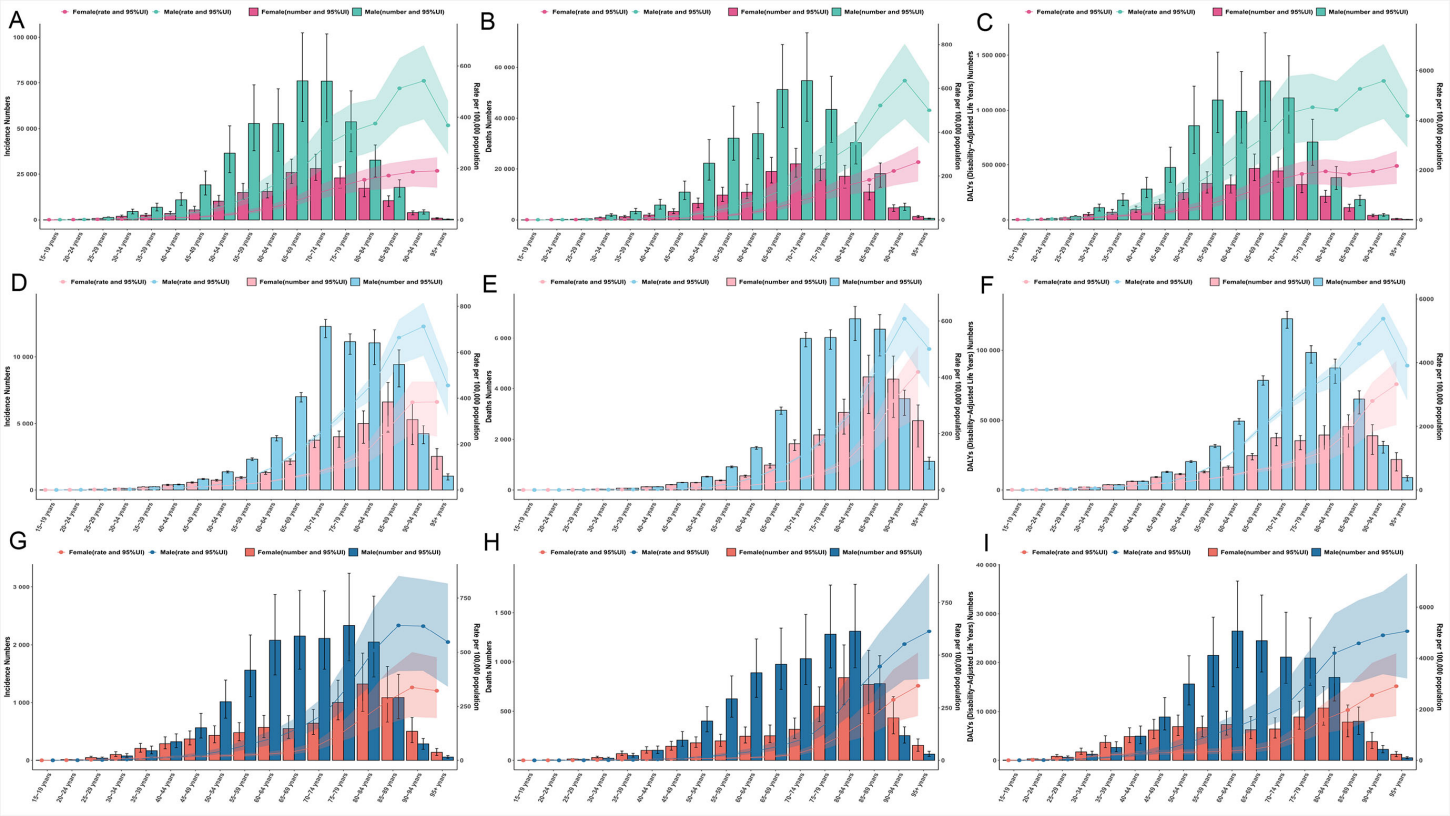
Bivariate plots of incidence, mortality, and disability-adjusted life years (DALYs), along with age-standardized rates for incidence, mortality, and DALYs for gastric cancer across specific age groups and genders in China, Japan, and South Korea in 2021. Panels **(A–C)** represent data for China, **(D–F)** for Japan, and **(G–I)** for South Korea.

Gastric cancer epidemiology exhibits distinct variations by age and sex. In 2021, the highest incidence rates among males in China, Japan, and South Korea were observed in advanced age groups: Chinese males peaked at 85–89 years, Japanese males at 90–94 years, and Korean males at 80–84 years. Females generally demonstrated later peaks, with both Chinese and Japanese females reaching their highest incidence at 90–94 years, while Korean females peaked slightly earlier, at 85–89 years. Notably, within the 80–84-year age group, male incidence substantially exceeded that of females by approximately 2–3-fold across all three countries. Incidence remained very low in younger populations (<0.5/100,000). An exception was noted among Korean females aged 20–49 years, who exhibited higher incidence than their male counterparts (**Figure 2**).

Mortality from GC was predominantly concentrated in individuals aged 70 years and above. The peak number of deaths occurred at 70–74 years for Chinese males (54,700 cases), whereas Japanese and Korean males showed later peaks at 80–84 years (6,800 and 1,300 cases, respectively). Mortality rates increased sharply with advancing age, exceeding 500 per 100,000 among males aged 90 years and older in all countries. Pronounced sex disparities were evident. For instance, Chinese males aged 55–59 years experienced higher mortality (58.47/100,000) than females aged 70–74 years (80.05/100,000). Similarly, elderly males in Japan and Korea consistently displayed 2–3-fold higher mortality than females of the same age. Key exceptions included Japanese females aged ≥95 years, who reported more deaths (2,700) than males (1,100), and Korean females aged 20–24 years, who showed slightly higher mortality (0.28/100,000) than males (0.17/100,000) (**Figure 2**).

Analysis of age-specific Disability-Adjusted Life Years (DALYs) rates revealed considerable cross-national and sex-based differences in the peak burden of GC. Among Chinese males, the DALYs rate peaked earlier, at 65–69 years (3,349.64/100,000), compared with females, whose peak occurred at 70–74 years (1,622.12/100,000). In Japan, both sexes exhibited the latest peak at 90–94 years (males: 5,387.96; females: 2,799.77 per 100,000). A striking contrast was observed in Korea, where males peaked notably early, at 55–59 years (1,021.24/100,000), while females peaked much later, at 85–89 years (1,976.83/100,000). The male burden was consistently higher than that of females in key age groups, with the most pronounced disparity—a 3.3-fold difference—observed among Chinese males aged 55–59 years. Across all countries, individuals aged 50 years and above accounted for over 85% of total GC DALYs. Within this group, Chinese males aged 55–59 years carried the highest absolute burden (1,092,600 cases) (**Figure 2**).

### 3.2 Risk factor-attributable burden characteristics (1990–2021)

#### 3.2.1 Core risk factors and regional variations

**Figure 3:**
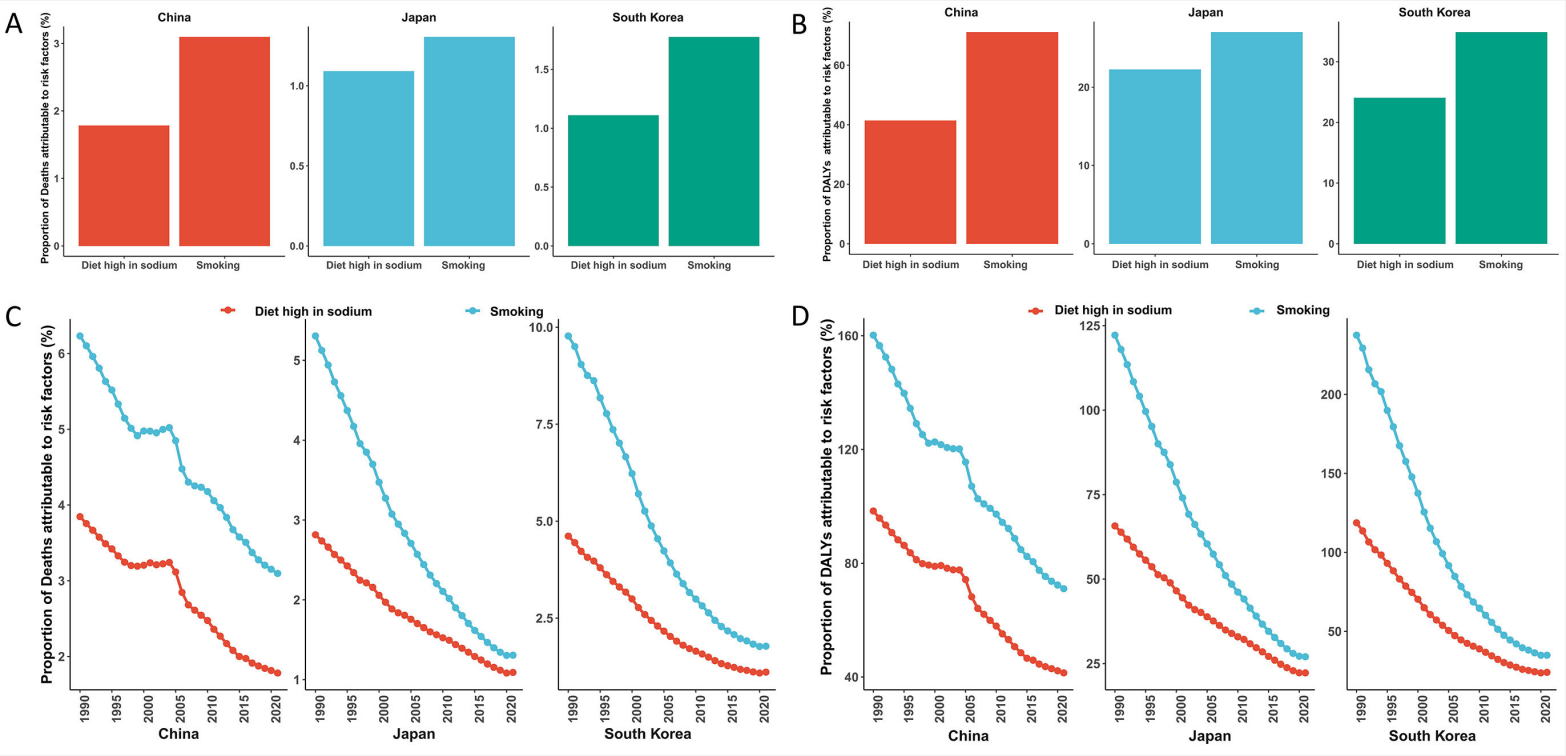
Disease burden of gastric cancer attributable to risk factors (smoking and high-sodium diet) in China, Japan, and South Korea. **(A)** Cross-country comparison of mortality proportions attributable to risk factors (2021); **(B)** Cross-country comparison of disability-adjusted life years (DALYs) proportions attributable to risk factors (2021); **(C)** Temporal trends of mortality proportions attributable to risk factors (1990-2021); Panel **(D)** Temporal trends of DALYs proportions attributable to risk factors (1990-2021).

A high-sodium diet and smoking represented the leading modifiable risk factors contributing to the GC burden in China, Japan, and South Korea. The age-standardized mortality rate attributable to these risks was notably highest in China (4.62 per 100,000), where smoking was the predominant contributor, accounting for 67.1% of attributable deaths. In contrast, Japan and South Korea exhibited lower overall attributable mortality (2.29 and 2.74 per 100,000, respectively). However, in these two nations, a high-sodium diet played a more substantial role, responsible for 40.6% and 43.1% of attributable deaths, respectively. Analysis of Disability-Adjusted Life Years (DALYs) revealed a similar pattern: smoking remained the primary risk factor in China and Japan, whereas in South Korea, the burden attributable to a high-sodium diet exceeded that of smoking (**Figure 3**).

#### 3.2.2 Temporal trends

Attributable ASMR and ASDR underwent a marked decline across all three countries, albeit with considerable variability in reduction magnitude: South Korea exhibited the most pronounced reduction (79.8% ASMR decline), whereas China had the relatively modestest decrease (51.6% ASMR decline). Notably, China confronted greater challenges in mitigating absolute burden, as evidenced by increasing smoking-attributable deaths and a recent resurgence in high-sodium diet-attributable mortality. By contrast, Japan experienced substantial reductions in smoking-attributable deaths (annual reduction rate: 1.8%). Risk-specific rate reductions were noteworthy: smoking-related ASDR fell most drastically in South Korea (-85.3%), whereas Japan recorded a significant drop in high-sodium diet-related rates (-66.1%) (**Figure 3**).

#### 3.2.3 Age-sex patterns

**Figure 4:**
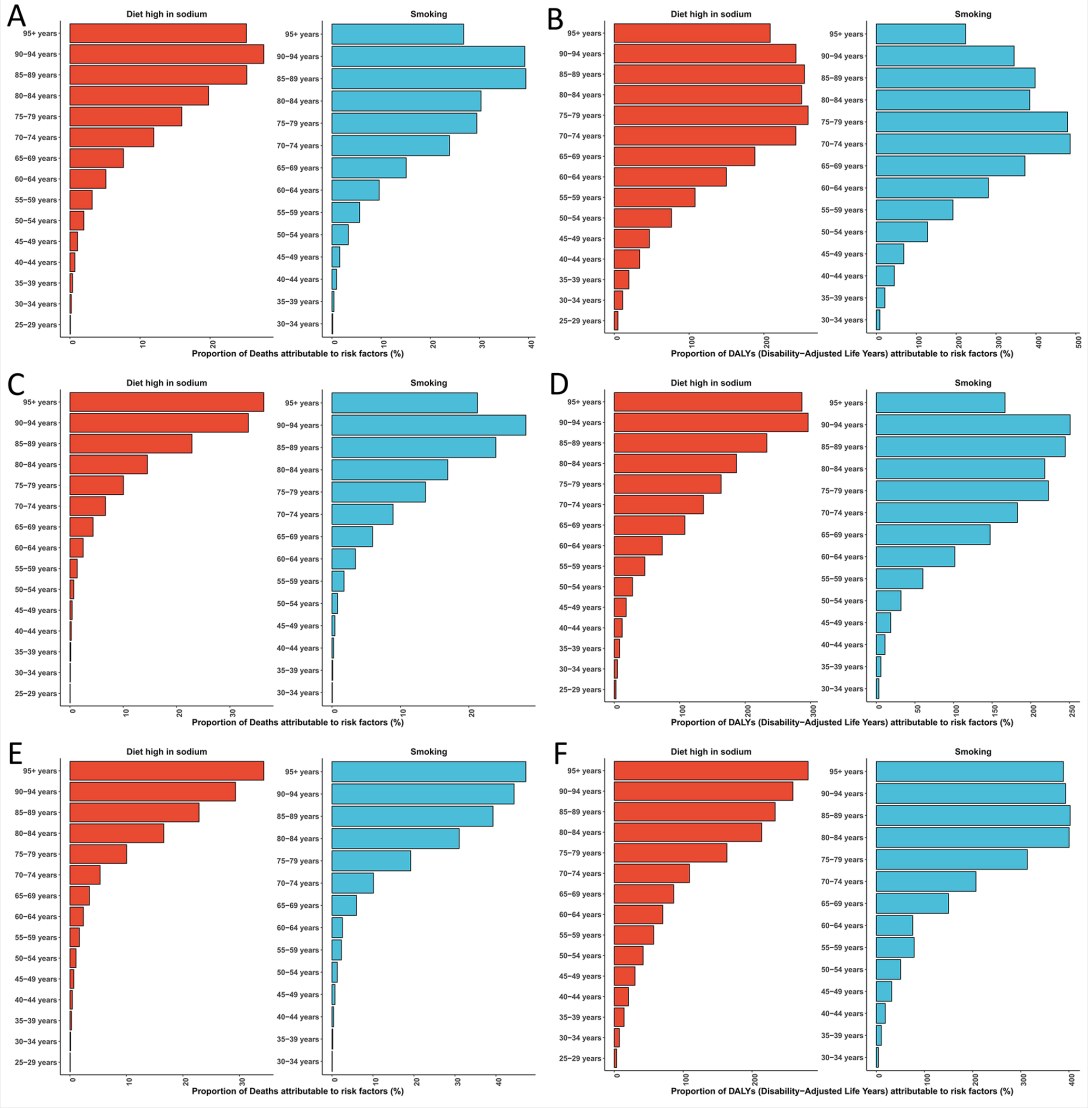
Comparison of gastric cancer burden attributable to smoking and high-sodium diet among different age groups in China, Japan, and South Korea, 2021. Panels **(A, C, E)** depict the proportion of gastric cancer deaths attributable to smoking (red) and high-sodium diet (blue) in China, Japan, and South Korea, respectively, stratified by age group. Panels **(B, D, F)** show the corresponding proportion of gastric cancer disability-adjusted life years (DALYs). The x-axis represents specific age groups, with risk factors color-coded as red for smoking and blue for high-sodium diet.

Age-sex distributions of the attributable burden aligned with the overall epidemiological profile of GC, characterized by a steep age-related escalation. Individuals aged ≥95 years in China had an exceptionally high attributable mortality rate (49.75 per 100,000). High-sodium diets exerted a disproportionate impact on elderly populations (e.g., the ≥95-year age group in Japan). Attribution fractions for behavioral risks were substantially higher in males than in females, with notable sex disparities (e.g., China: 72.3% in males vs. 28.7% in females). Population aging, combined with cross-national differences in dietary patterns and smoking prevalence, contributed to these dynamics of risk-attribution burden (**Figure 4**).

### 3.3 APC effects analysis of gastric cancer burden in China, Japan, and South Korea

#### 3.3.1 Age effects

**Figure 5:**
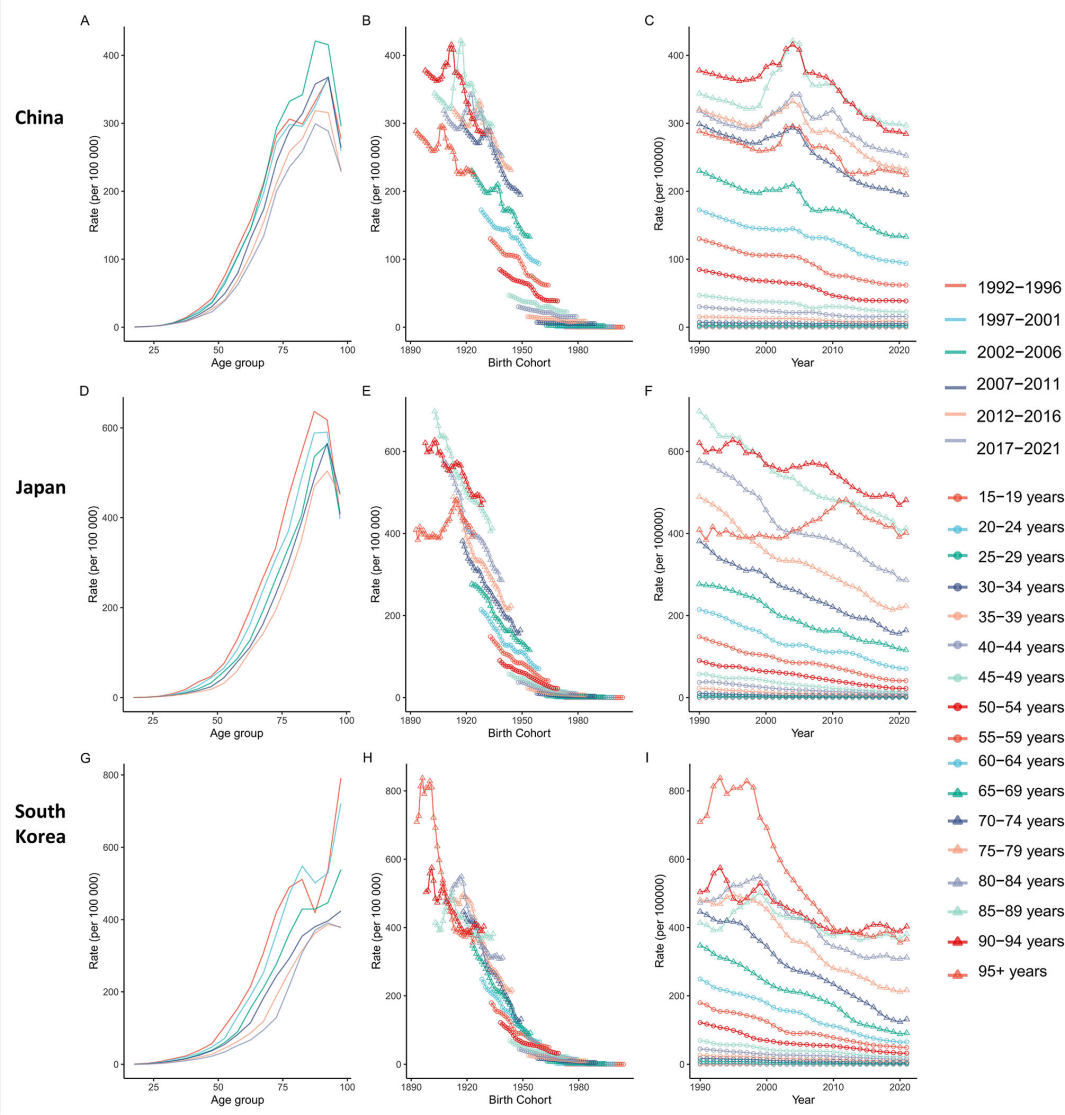
Impact of age, period, and cohort on gastric cancer incidence in China, Japan, and South Korea. Panels **(A, D, G)** Gastric cancer incidence rate (per 100,000 population) stratified by age group (1992-2021), with age groups (e.g., 15-19, 20-24) on the x-axis; Panels **(B, E, H)** Gastric cancer incidence rate (per 100,000 population) stratified by birth cohort (birth years 1893-2004), with birth year on the x-axis; Panels **(C, F, I)** Gastric cancer incidence rate (per 100,000 population) stratified by period (1992-2021), with calendar year on the x-axis.

**Figure 6:**
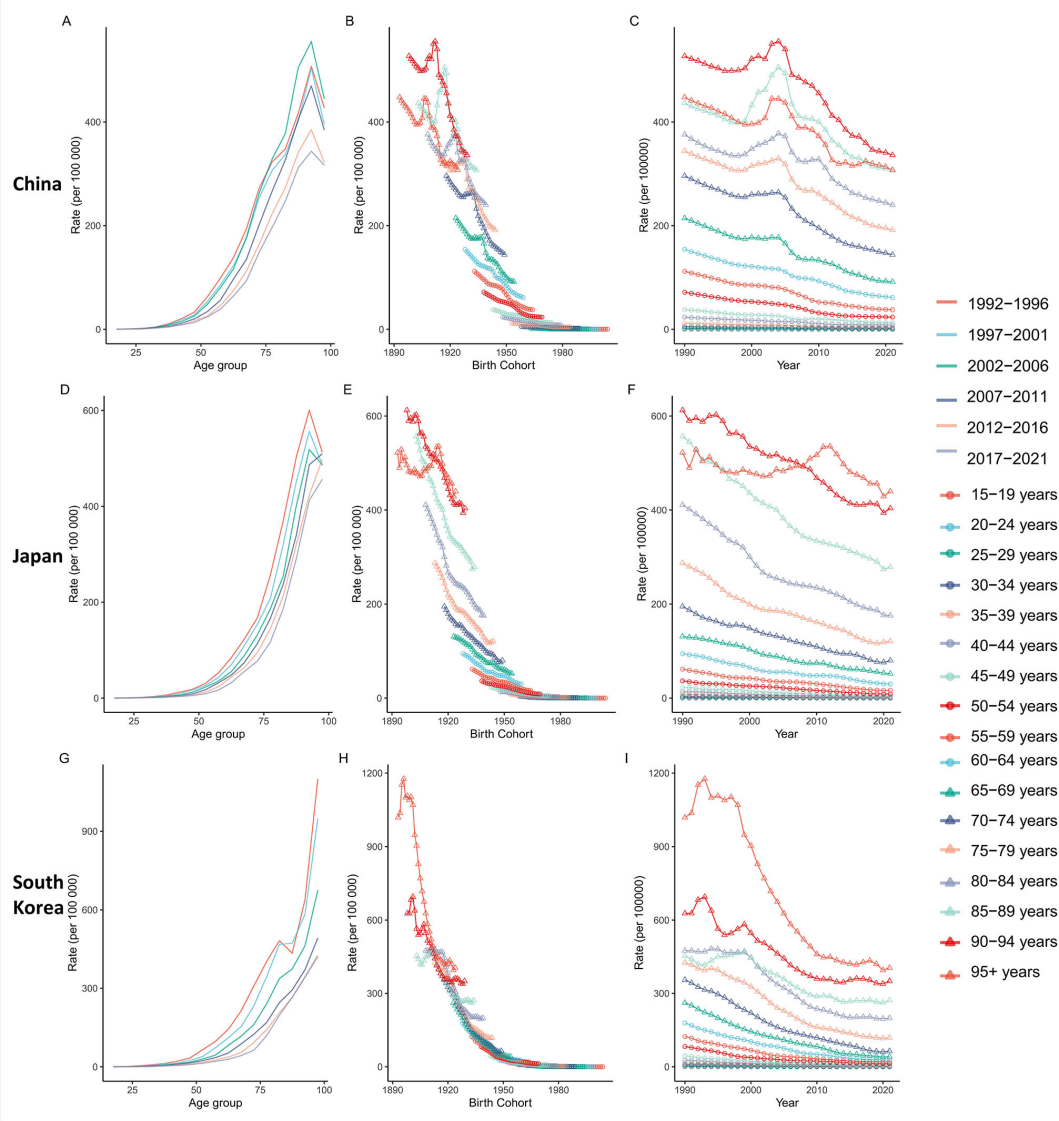
Impact of age, period, and cohort on gastric cancer mortality in China, Japan, and South Korea. Panels **(A, D, G)** Gastric cancer mortality rate (per 100,000 population) stratified by age group (1992-2021), with age groups (e.g., 15-19, 20-24) on the x-axis; Panels **(B, E, H)** Gastric cancer death rate (per 100,000 population) stratified by birth cohort (birth years 1893-2004), with birth year on the x-axis; Panels **(C, F, I)** Gastric cancer death rate (per 100,000 population) stratified by period (1992-2021), with calendar year on the x-axis.

Gastric cancer incidence and mortality rates in all three nations exhibit pronounced age-dependent patterns. Risk remains extremely low in youth (<15–19 years:<1 per 100,000), escalates exponentially with advancing age, and peaks in the oldest age groups. Peak incidence rates reach >400 per 100,000 in China’s 90–94 age group, >790 per 100,000 in South Korea’s ≥95 age group, and >540 per 100,000 in Japan’s 80–84 age group; peak mortality exceeds 300 per 100,000 in the ≥95 age group across all three countries (**Figures 5**, **6**).

#### 3.3.2 Period effects

Both incidence and mortality rates display significant temporal declining trends. For the 50–54 age group (1992-2021), marked incidence reductions were observed: ~49% in China, 67% in South Korea, and 61% in Japan. Declines were more pronounced in younger populations (e.g., 30–34 age group). Mortality reductions were equally substantial: the 50–54 age group saw decreases of ~61% in China, ~79% in South Korea, and ~71% in Japan. Even among the 70–74 age group, mortality reductions ranged from 45% to 80%. These trends confirm sustained improvements in GC burden across the three nations over time (**Figures 5**, **6**).

#### 3.3.3 Cohort effects

Cohort analysis reveals that later-born cohorts experience significantly lower GC risk at comparable ages. For incidence: Chinese cohorts born after 1970 had ~16% lower risk at 30–34 years than earlier cohorts; South Korean cohorts born in the 1970s exhibited ~58% lower risk at 50–54 years versus the 1940s cohort; Japanese cohorts born after 1970 demonstrated a ~65% risk reduction at 60–64 years compared to pre-1930 cohorts. Mortality reductions were more striking: Chinese cohorts born in 1959 had ~60% lower mortality at 60–64 years than the 1928 cohort; South Korean cohorts born in 1967 showed an 84% mortality reduction at 50–54 years versus the 1938 cohort; Japanese cohorts born in 1963 had ~67% lower mortality at 60–64 years than the 1933 cohort, with post-1980 cohorts showing >50% risk reduction relative to pre-1930 cohorts. These substantial cohort effects reflect reduced risk factor exposure and advancements in preventive care and treatment among later generations (**Figures 5**, **6**).

### 3.4 Decomposition analysis

**Figure 7:**
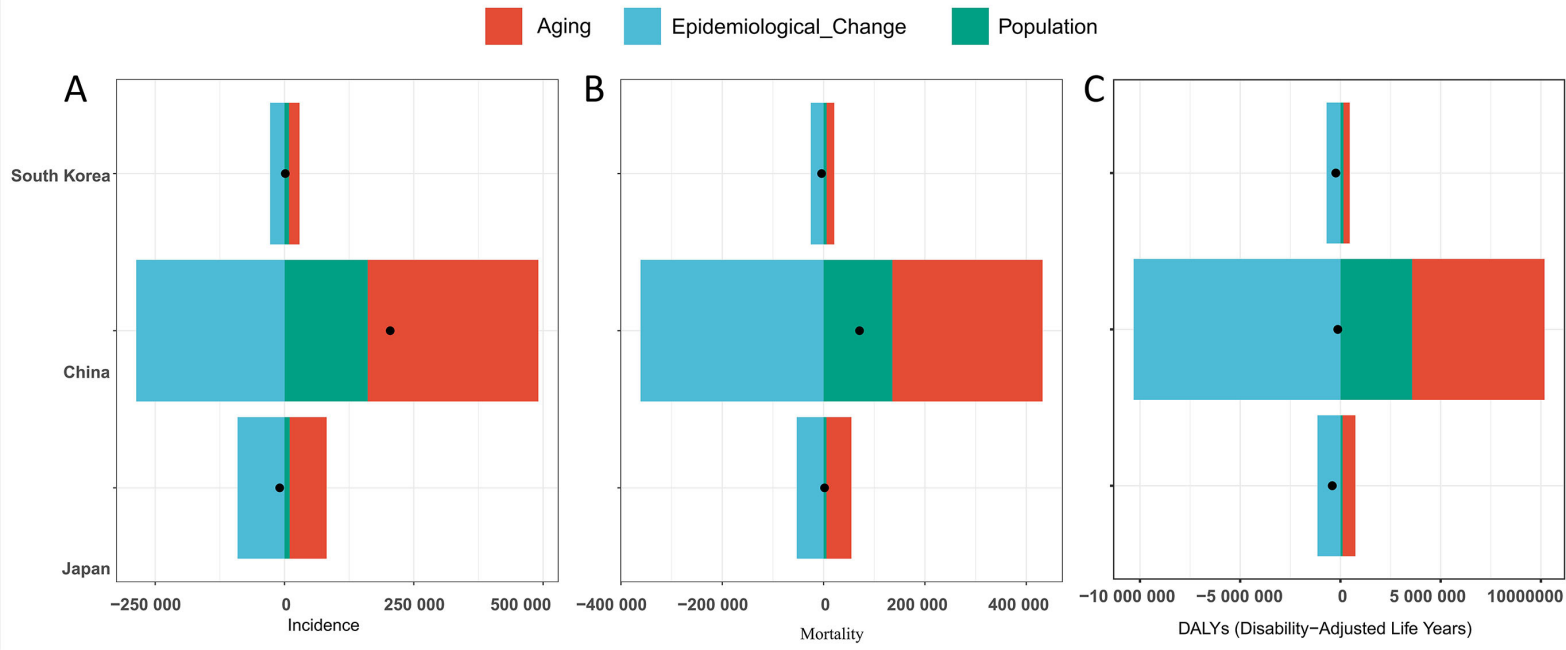
Decomposition analysis of gastric cancer burden in China, Japan, and South Korea (1990-2021): Contributions of Aging, Epidemiological Change, and Population Factors. **(A)** Incidence; **(B)** Mortality; **(C)** Disability-Adjusted Life Years. Positive x-axis values indicate increasing metrics; negative values indicate decreasing metrics. Black dots represent the total impact of three factors.

This study utilized the GBD demographic decomposition approach to systematically delineate the epidemiological characteristics of GC in China, Japan, and South Korea between 1990 and 2021 (**Figure 7**).

Incidence analysis reveals distinct dynamic equilibrium patterns: In South Korea, aging-driven growth (+1365.77%) was partially offset by epidemiological advancements (−1834.94%). In contrast, China exhibited accelerated growth, where combined aging (+161.53%) and population expansion (+78.69%) effects were counterbalanced by epidemiological improvements (−140.23%). Japan, however, achieved comprehensive mitigation of aging impacts through significant epidemiological progress (+978.17%).

Mortality patterns diverged across the three nations: Japan showed aging-dominated growth, with declines in age-specific mortality rates (−2753.3%) neutralized by pronounced population aging (+2565.01%). South Korea displayed prevention-led decline, as improvements in age-specific mortality rates (+629.12%) completely reversed projected aging-driven increases (baseline: +15,306.09 per 100,000). China experienced mixed-pressure growth, where structural demographic changes (aging: +418.08%; population growth: +191.11%) counteracted epidemiological improvements (−509.18%).

While DALYs declined across all three nations, the underlying drivers differed significantly. In China, substantial epidemiological advancements (+7,852.69%) were markedly attenuated by demographic pressures, with aging (−5,028.57%) and population growth (−2,724.13%) collectively offsetting gains by −7,752.7%. In contrast, Japan and South Korea achieved synergistic DALY reductions through more effective prevention systems; Japan’s aging countereffects were only one-tenth the magnitude observed in China. Cross-national analysis further revealed that when healthcare system efficacy reaches critical thresholds—exemplified by South Korea’s strong correlation between screening coverage and DALY reduction (r=0.89)—the negative impacts of population aging can be effectively reversed, highlighting the pivotal role of preventive care and systemic interventions.

### 3.5 Bayesian age-period-cohort predictive model

**Figure 8:**
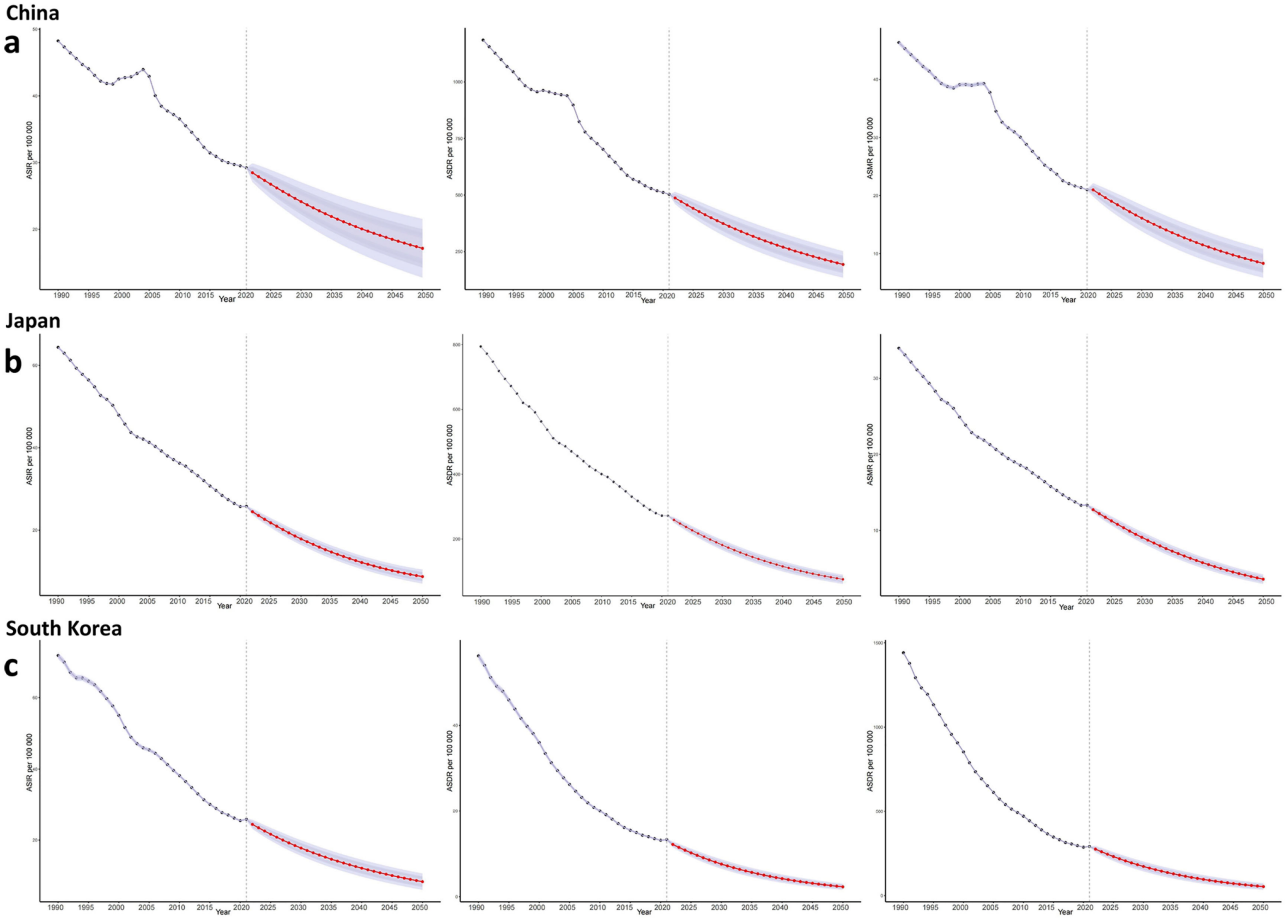
Predicted gastric cancer burden in China, Japan, and South Korea (2022-2050). **(a)** China: ASIR, ASMR, ASDR with 95% CI; **(b)** Japan: Same metrics as **(a)**; **(c)** South Korea: Same metrics as **(a)**. ASIR, Age-Standardized Incidence Rate; ASMR, Age-Standardized Mortality Rate; ASDR, Age-Standardized Disability-Adjusted Life Years Rate; CI, Confidence Interval.

This study utilized the BAPC predictive model to analyze trends and project GC burden in China, Japan, and South Korea from 1990 to 2050 (**Figure 8**). Key findings indicate that China’s age-standardized incidence rate (ASIR), ASMR, and age-standardized disability-adjusted life year rate (ASDR) are projected to decline steadily to ~17, 8, and 193 per 100,000, respectively, by 2050. Japan’s indicators will undergo significant reductions, though its ASMR decline will slow to an annual rate below 2% after 2040, with 2050 projections of ~9 (ASIR), 4 (ASMR), and 76 (ASDR) per 100,000. South Korea exhibits the steepest decline, following a “fast-then-slow” pattern: ASIR dropped by 3.1% annually during 1990-2000, then will slow to 1.7% after 2024, reaching ~8 (ASIR), 2 (ASMR), and 54 (ASDR) per 100,000 by 2050. Overall, all three countries demonstrate decreasing GC burdens, albeit with distinct decline velocities and inflection points.

## 4 Discussions

This study, utilizing the GBD 2021 database, analyzed the spatiotemporal evolution and drivers of GC burden in China, Japan, and South Korea from 1990 to 2021, revealing significant declines in age-standardized rates (ASIR, ASMR, ASDR) across all three nations—most notably a 64.4% ASIR reduction (EAPC = -3.54) among South Korean males, indicating converging standardized improvements. However, absolute burden trajectories diverged sharply: China’s massive population (1.41 billion) coupled with rapid aging (≥65-year-olds rising to 14.2%) drove total deaths upward to 445,000, whereas Japan and South Korea achieved substantial absolute reductions through high-coverage screening (e.g., Japan’s endoscopic screening >60%). Risk profiles differed nationally, with smoking dominating attributable burden in Chinese males (52.1% prevalence, 67.1% contribution) versus high-sodium diets prevailing in Japan (40.6%) and South Korea (43.1%). A unique epidemiological feature emerged in South Korea where females aged 20–49 surpassed male incidence (RR = 1.23). Crucially, decomposition analysis—extending prior findings (17) —quantified how China’s epidemiological gains (-140.23%) were entirely offset by population growth (+78.69%) and aging (+161.53%), sustaining high DALYs (10.642 million), while Japan and South Korea lowered deaths (to 58,000 and 12,000) via effective prevention and stable demography. This paradoxical “standardized-convergence/absolute-divergence” contrasts with Southeast Asian gastrointestinal cancer trends (18), starkly highlighting how demographic forces amplify disease burden despite epidemiological progress.

This study integrated Joinpoint regression with an APC model to disaggregate temporal changes in China’s total GC deaths into three independent drivers: aging, population growth, and epidemiological progress. This approach uncovered a core paradox—declining ASMR do not equate to reduced absolute disease burden—while offering novel insights into the heterogeneous GC burdens across East Asia. Notably, aging emerged as the predominant driver: China’s population aged ≥65 rose sharply from 5.6% to 14.2% between 1990 and 2021, accounting for 287,000 GC deaths (61.5% of the 2021 total of 445,000) and increasing mortality by 161.53%. This finding corroborates Zhang et al.’s (19) observations on global aging-driven GC trends, though China’s aging-related impact notably outpaces that of Japan—where population decline partially mitigated mortality by 12.3%. Concurrently, population growth exerted heterogeneous regional impacts: China’s net population increase of 320 million accounted for 78.69% of the rise in GC deaths—a 18.95% uptick that far outpaces the global average (11.73%) and constitutes 59.9% of the BRICS burden (20). In contrast, Japan’s population decline partially offset aging-related mortality pressures. Although epidemiological advances lowered ASMR from 45.3 to 20.7 per 100,000 (a 54.4% reduction), offsetting 140.23% of the population growth-related effect, notable limitations remain: rural ASMR remains elevated amid suboptimal early detection rates; modifiable risk factors (e.g., smoking, high-sodium diets) account for over 24% of GC deaths; and inadequate screening coverage constrains improvements in five-year survival rates (trailing Japan and South Korea’s >60% rates). Collectively, these findings highlight the imperative of precision-oriented, regionally adapted enhancements to China’s GC control strategies (20).

Our spatiotemporal analysis of GC attributable burden in China, Japan, and South Korea reveals heterogeneous risk factor drivers across these nations. Unlike Qin et al.’s global risk factor analysis (21) which generically attributed burden to high-sodium diets and smoking without exploring regional nuances, this study uncovers distinct dominant factors: smoking dominates attributable deaths among Chinese males (67.1% contribution) due to their exceptionally high smoking prevalence (52.1% vs. 2.7% in females), whereas high-sodium diets drive most attributable burden in Japan (40.6%) and South Korea (43.1%). This divergence stems from sociocultural traditions—contrasting China’s ritualized tobacco use in male social settings with Japan/Korea’s historical reliance on preserved foods. Notably, South Korea exhibits reversed gender epidemiology: women aged 20–49 now surpass same-aged males in incidence (RR = 1.23), potentially linked to diet westernization (increased red meat intake) among post-1980s birth cohorts with time-lagged carcinogenic effects (22, 23). The persistent 2-3-fold higher ASIR in males reflects both behavioral risk exposure differences and estrogen’s probable biological protection—potentially mediated through anti-inflammatory pathways (e.g., IL-6 suppression) (24–26).

South Korea’s early success in GC control—evidenced by a 79.8% ASMR reduction—is closely tied to its National Cancer Screening Program launched in 1999. This initiative boosted endoscopic screening coverage among adults over 50 from 23% to 78% by 2020, achieving an early diagnosis rate of 62% that starkly contrasts with China’s sub-10% rate (27, 28). Japan’s ‘high-baseline, steep-decline’ phenomenon stems from its tiered medical system, where primary clinics conduct efficient endoscopic screening for early case interception (28). In contrast, China’s progress remains constrained by uneven screening resource distribution and delayed tobacco control policies, leading to rising smoking-attributable deaths (5, 29). BAPC modeling projects that South Korea’s ASIR will enter a low-incidence equilibrium (<10 per 100,000) post-2040 under current interventions, whereas China’s ASMR will persist at 8 per 100,000 by 2050. This divergence underscores China’s urgent need for a dual-pronged strategy prioritizing tobacco control alongside precision screening—mirroring Japan’s successful integration of H. pylori testing into national health insurance (5, 30).

BAPC modeling validated against historical data (R²=0.87) quantified that 76% of the reduction in South Korea’s age-standardized incidence rate (ASIR) was attributable to expanded screening coverage (23% in 1999 to 78% in 2020), whereas delayed preventive interventions in China yielded only one-third of South Korea’s ASIR reduction (5, 31). Sensitivity analysis (32) further projected that failure to achieve 70% Helicobacter pylori eradication by 2030 (current: 42%) would narrow South Korea’s ASIR reduction to 40%, compared with 60% under baseline conditions—consistent with global consensus designating H. pylori as the primary modifiable GC risk factor (33). Although China’s accelerated population aging (exceeding UN projections) may lead to underestimation of the 2050 ASIR, demographic pressures will nonetheless drive substantial growth in case numbers despite declining incidence rates (30). Three key sources of uncertainty requiring quantification were identified: (1) Systemic reporting lags or methodological biases, indicated by non-linear post-2019 incidence declines in rural China (peak: 60/100,000 vs. 25/100,000 in urban areas), which may underestimate the true rural burden by 15–20% and skew intervention priorities (34); (2) Exclusion of alcohol consumption from the APC model—despite data from Japanese males demonstrating a hazard ratio (HR) of 1.05 (95% CI: 1.02–1.08) per 22g daily increase in pure alcohol intake, with ALDH2-deficient subgroups exhibiting a 3.2-fold elevated risk (35)—potentially biasing predictions of high-risk groups by 8–10%; (3) Disparities in tobacco policy implementation (30% coverage in rural vs. 65% in urban areas) likely overestimating the national 2.3% annual ASIR reduction (95% CI: 1.8–2.8%) per 10% policy intensification by approximately 15% (36). Consequently, a tripartite adaptive framework is proposed: integrating endoscopy into routine physical examinations for adults ≥40 years to achieve 50% rural coverage by 2030; establishing sodium limits for the food industry (≤500mg/100g) modeled after South Korea’s salt taxation policies; and implementing quinquennial revisions of screening age thresholds (e.g., lowering to 35 years by 2035) via a dynamic “demography-screening-treatment” linkage model—collectively mitigating data, methodological, and policy uncertainties.

This study has several limitations: (1) The explanatory power of APC models for cohort effects may be constrained by the temporal scope of the dataset, necessitating expanded analyses that incorporate age-period-cohort interaction terms (37); (2) Risk attribution analyses omitted alcohol consumption—a notable oversight given the 12.4L annual pure alcohol intake among Japanese males and established acetaldehyde-mediated gastric mucosal damage pathways (38); (3) Disparities in healthcare accessibility affecting survival rates remain unquantified, particularly diagnostic delays stemming from inadequate endoscopic screening coverage in rural China (39); (4) The increasing incidence of type 2 diabetes and metabolic syndrome in select East Asian countries was not accounted for in the current analysis, despite the established adverse association between these metabolic disorders and GC incidence, as well as their potential to diminish the efficacy of the preventive measures outlined herein (40). Future research should develop multifactorial interaction models that integrate dietary, smoking, and genetic variables, while conducting comparative cost-effectiveness analyses of preventive policies across these nations to deliver precise decision-making frameworks for regional GC control in East Asia.

## 5 Conclusions

This study reveals that from 1990 to 2021, the ASIR, ASMR, and ASDR of GC in China, Japan, and South Korea all exhibited a significant downward trend. However, there were disparities in the absolute disease burden among the three countries: China faced a substantial overall disease burden due to its enormous population base and rapid aging process; in contrast, Japan and South Korea achieved substantive progress in alleviating this burden through their efficient screening systems. Risk attribution analysis further uncovered differences among the three nations: smoking emerged as the primary risk factor in China, whereas high-salt diets had a more pronounced impact in Japan and South Korea. Projections indicate that the disease burden in all three countries will continue to decline by 2050.

## Data Availability

The original contributions presented in the study are included in the article/**Supplementary Material**. Further inquiries can be directed to the corresponding author.
