# Supplementary material for: Comparative analysis of gastric cancer risk attribution (1990-2021) and 2050 burden projection in China, Japan, and South Korea: an age-period-cohort modeling approach based on the Global Burden of Disease 2021 study
Source: Front Oncol. 2026 Jan 21;15:1680684. doi: 10.3389/fonc.2025.1680684 (PMC12869315; doi:10.3389/fonc.2025.1680684)
Supplement: Supplementary file 1 [file DataSheet1.docx]

**
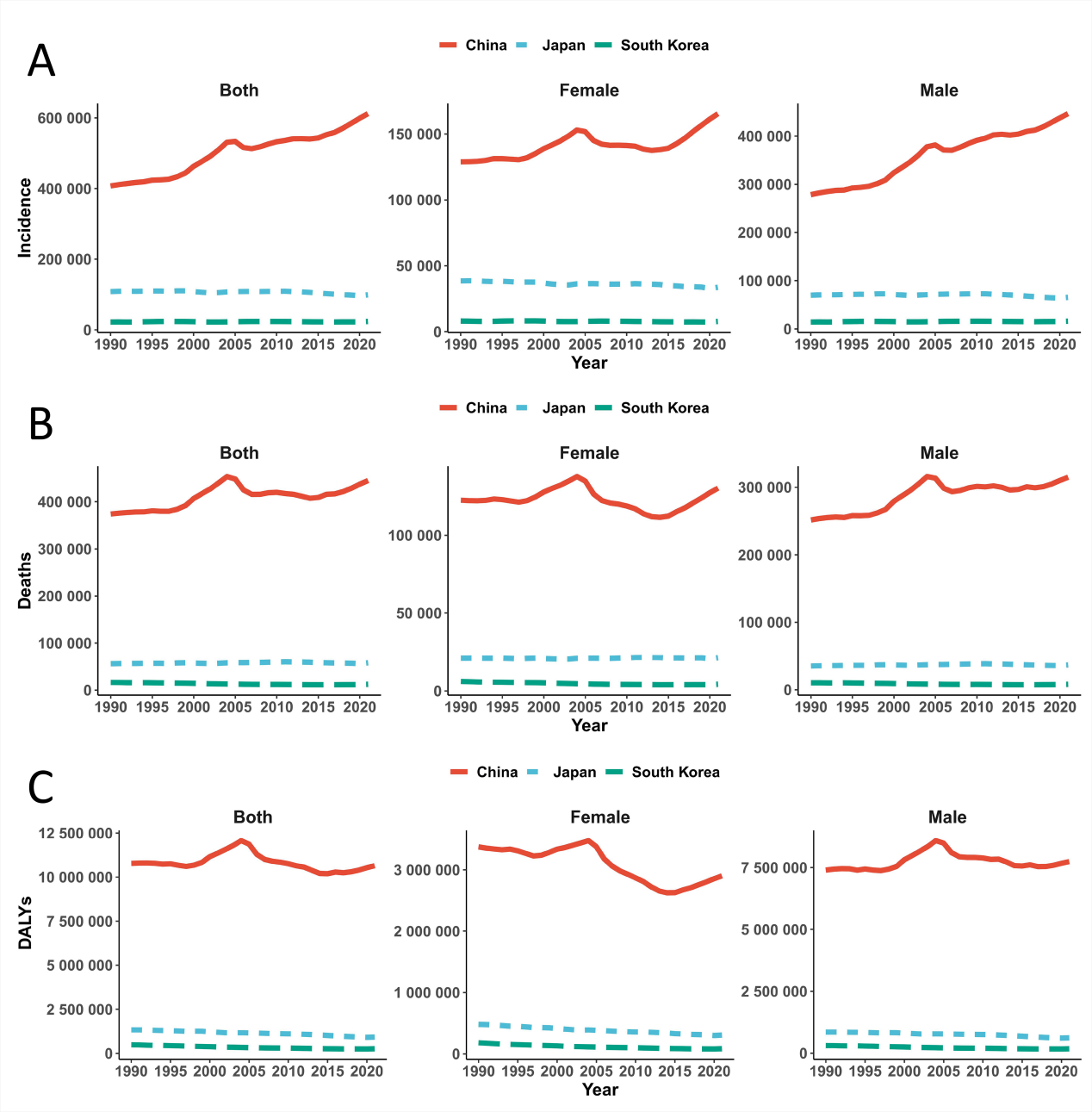
**

**Supplementary Figure 1**. Trends in gastric cancer case counts incidence (A), deaths (B) and DALYs (C) in China, Japan, and South Korea (1990–2021), stratified by sex. DALYs, disability-adjusted life years.
